# The greatest risk for low-back pain among newly educated female health care workers; body weight or physical work load?

**DOI:** 10.1186/1471-2474-13-87

**Published:** 2012-06-06

**Authors:** Jette Nygaard Jensen, Andreas Holtermann, Thomas Clausen, Ole Steen Mortensen, Isabella Gomes Carneiro, Lars Louis Andersen

**Affiliations:** 1 National Research Centre for the Working Environment, Lersø Parkalle 105, DK 2100, Copenhagen, Denmark; 2 Department of Occupational and Environmental Medicine, Bispebjerg University Hospital, Bispebjerg Bakke, DK 2400, Copenhagen, Denmark

**Keywords:** Prospective cohort study, Low back pain, Physical work load, Health care work, Musculoskeletal disorders, Body mass index

## Abstract

**Background:**

Low back pain (LBP) represents a major socioeconomic burden for the Western societies. Both life-style and work-related factors may cause low back pain. Prospective cohort studies assessing risk factors among individuals without prior history of low back pain are lacking. This aim of this study was to determine risk factors for developing low back pain (LBP) among health care workers.

**Methods:**

Prospective cohort study with 2,235 newly educated female health care workers without prior history of LBP. Risk factors and incidence of LBP were assessed at one and two years after graduation.

**Results:**

Multinomial logistic regression analyses adjusted for age, smoking, and psychosocial factors showed that workers with high physical work load had higher risk for developing LBP than workers with low physical work load (OR 1.8; 95% CI 1.1–2.8). In contrast, workers with high BMI were not at a higher risk for developing LBP than workers with a normal BMI.

**Conclusion:**

Preventive initiatives for LBP among health care workers ought to focus on reducing high physical work loads rather than lowering excessive body weight.

## Background

Low back pain (LBP) represents a major socioeconomic burden for the Western societies [1]‐[2] with considerable individual consequences in terms of morbidity and disability[3]. Chronic LBP is also a risk factor for developing knee pain [4]. Health care workers show a higher prevalence of LBP [5] than many other occupational groups [6]‐[8]. The annual prevalence of LBP among health care workers is as high as 77% [8].

Health care workers are generally characterized by having a high physical work load and high prevalence of overweight [9]‐[10]. However, a causal relationship between these risk factors and development of LBP remains debatable. A main reason for the debate is the different methodological designs of previous studies. For example, several studies have included health care workers with existing LBP in their analyses [11-13]. Given that studies have shown that earlier history of LBP is a strong predictor of future LBP [14]‐[15], this may have contributed to the inconsistent findings. Other methodological aspects, like cross-sectional designs and study populations with varying durations of exposure to physical work demands at baseline may also be related to different conclusions. Thus, prospective cohort studies determining the risk for developing LBP due to being overweight or obese and to have moderate or high physical work load are necessary for optimizing preventative strategies.

This prospective cohort study determines the risk of developing LBP from moderate or high physical work loads and from being overweight or obese among newly educated female health care workers without prior history of LBP.

## Methods

### Study design

Data used in this prospective study was from cohort study of all newly educated health care helpers and assistants in Denmark—the Danish Health Care Worker Cohort-Class of 2004 (DHCWC-2004) [16]. Health care helpers undergo a 14 months training and they are qualified for work with nursing and practical assistant in eldercare. Health care assistant training builds on the previous training for health care helpers and requires an additional 20 months of training. Assistants are qualified for work in eldercare, as well as hospitals, and their work is related to coordination and teaching activities, along with basic health and nursing activities. The DHCWC-2004 cohort consisted of a baseline questionnaire applied a few weeks before the students finished their education, a follow-up one year later (first follow-up) and a final follow-up two years later (second follow-up). The questionnaires applied were quite similar, with changes from baseline to first follow-up to adjust to the change from the training environment in baseline to the work environment. From first follow-up to second follow-up the questions were the same. This study consists of data from baseline (2004), one-year-after follow-up (2005) and two-year-after follow-up (2006). In this study, health care helpers and assistants are referred to as health care workers.

**Figure 1:**
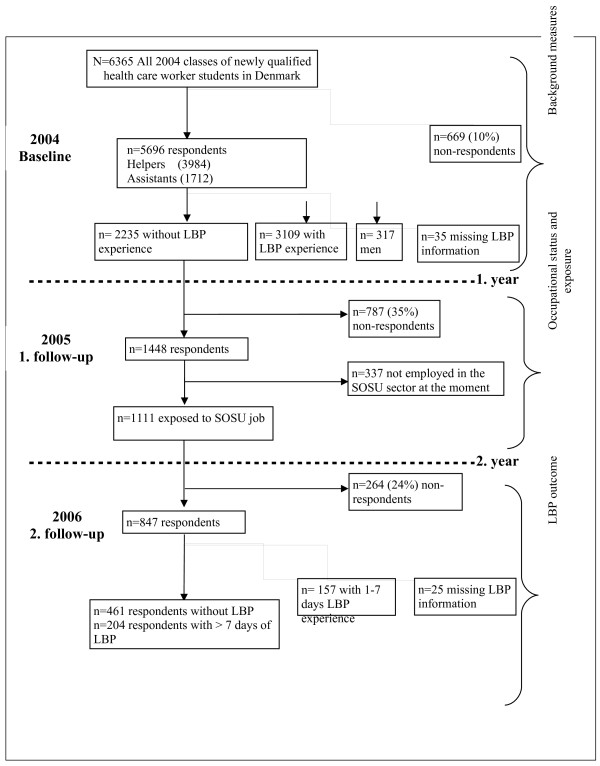
Flowchart of the study population.

Figure 1 presents the flow of participants through the cohort study. In short, 6,365 students were invited to participate in the baseline survey in 2004. Of those, 5,696 (90%) returned the baseline questionnaire. One and two years after graduation, data for the first and second follow-up questionnaires was gathered. The proportion of response rate in 2005 and 2006, were 65% and 54%, respectively.

Of the baseline respondents, 317 men were excluded from further analyses. For avoiding plausible influence of low back pain on the reporting of physical work demands, the health care workers reporting any previous or present low back pain at baseline (n = 3,109) were also excluded from further analyses. The study population at baseline consisted of 2,235 newly educated female health care workers without prior history of LBP. In the 1^st^. follow-up, after excluding non-respondents, as well as those without a job, in a job not involving health care, on long term sickness absence, or under continued education (n = 337), 1,111 respondents remained in the study population. Similarly, after exclusion of non-respondents and of those missing information on LBP in that round, the total number of respondents was 847.

### Ethical approval

The study has been notified to and registered by the Danish Data Protection Agency (Datatilsynet). According to Danish law, questionnaire and register based studies do not need approval by ethical and scientific committees, nor informed consent.

### Variables

The outcome variable was self-reported levels of LBP at the second follow-up. The information was collected by the following question from the Standardised Nordic Questionnaires (SNQ) for the analyses of musculoskeletal symptoms, which reliability has been shown to be acceptable [17]: ‘What is the total length of time that you have had low back pain during the last 12 months?’ As in the original SNQ, the location of the lower back was defined by a drawing with a marked area. Response categories were: ‘No LBP problem’; ‘1–7 days’; ‘8–30 days’; ‘more than 30 days, but not every day’; and ‘every day’. In order to perform the statistical analyses, that question was classified into ‘No LBP problem’, ‘Low back pain 1–7 days’ and ‘Low back pain over 7 days’ (the last three response categories grouped together).

Body mass index (kg/m^2^) was estimated from self-reported data on height and weight from first follow-up. Respondents were categorized according to the definitions of the World Health Organizations as normal weight (BMI = 18.5-24.9), overweight (BMI25.0-29.9) and obese (BMI over 30.0). Respondents with BMI < 18.5 were excluded from the analyses (n = 196).

The health care workers reported physical work load at the first follow-up with fourteen questions constituting the validated physical work load index by Hollmann [18]. Three items concerned postures of the trunk during lifting (time strongly inclined, twisted and laterally bent). Two items concerned positions of the arms (time with one arm above shoulder height, and two arms above shoulder height). Three items concerned the position of the legs (time with squatting. kneeling on one or both knees, walking or moving). Three items concerned lifting of weights with the trunk upright, and three items concerned lifting with trunk inclined 60 degrees (time with lifting light (up to 7 kg.), medium (8– 30 kg.) and heavy (more than 30 kg.) loads). The items were also presented as pictograms. The answers were given on a 5-point rating scale ranging from ‘never’ to ‘very often’. All the questions were transformed into the physical work load index aggregating all questions into a value representing the total load of the lumbar spine during work, which has been tested for validity and reliability and been found satisfactory [18]. Given no documented threshold for physical work load exists, we have trichotomized the index based on the frequency distribution of the questionnaire replies: ‘Low physical work load’ (index from 0–12.929); ‘Moderate physical work load’ (index from 12.930-21.000); and ‘High physical work load’ (index from 21.001-56.17).

Age was obtained from register data and was treated as a continuous variable. Smoking was assessed by asking ‘Do you smoke every day?’ with three response categories: (1) ‘Yes’, (2) ‘No, but I have smoked before’, (3) ‘No, I have never smoked’. Influence at work and social support at work were scales from 0–100, measured by the Copenhagen Psychosocial Questionnaire (COPSOQ) [19,20].

The experience of previous periods of LBP was reported by the following question in the baseline questionnaire: ‘Have you ever experienced low back pain (pain or discomfort)?’ Response categories were ‘Yes’ or ‘No’. This information was used in order to limit our study population to only those female health care workers that did not report previous LBP (n = 2,235).

### Statistical analysis

Multinomial logistic regressions were used for modelling the risk for LBP. The statistical models were specified a priori. In the first multinomial logistic regression model (Model I), the relationship between BMI and physical work load (measured in the first follow-up) with LBP (measured in the second follow-up) was analysed. In Model II, age, smoking, social support at work and influence at work (variables measured in first follow-up) were included as covariates. An alpha level of 5% was considered statistically significant. The statistical analyses were performed with IBM SPSS Statistics 20.

## Results

Figure 1 shows that, 42 percent of the respondents at baseline (N = 2,235) had never experienced LBP. Two years later among those without prior history of LBP at baseline, 56% (n = 461) had not experienced any LBP the previous 12 months, 19% (n = 157) had experienced 1–7 days of LBP, and 25% (n = 204) had experienced > 7 days of LBP.

Table 1 shows characteristics of the study population. Small, but statistically significant differences at first follow-up between respondents with low, moderate and high physical work load were observed for BMI, age, social support at work and influence at work. Significant differences at first follow-up between respondents with normal weight, overweight and obese were observed for age and influence at work.

**Table 1:** Characteristics at first follow-up of newly educated female health care workers without previous history of LBP at baseline (n = 2,235)

	Physical work demands				Body mass index			
	Low (n = 363)	Middle (n = 330)	High (n = 302)	P-value	Normal (n = 798)	Overweight (n = 382)	Obese (n = 175)	P-value
Physical work demand (Mean, SD^a^)	8.60 (2.86)	16.72 (2.19)	29.20 (6.76)	<0.001^*^	17.31 (9.55)	17.36 (9.38)	18.81 (9.24)	0.265
Body mass index (Mean, SD^a^)	24.66 (4.54)	25.73 (5.51)	24.91 (5.57)	0.022^*^	22.08 (1.70)	27.07 (1.39)	34.80 (6.08)	<0.001^*^
Age in years (Mean, SD)	36.90 (10.21)	35.58 (10.39)	34.44 (10.54)	0.010^*^	34.02 (10.79)	35.98 (10.56)	35.17 (9.76)	0.012^*^
Social support at work (Mean, SD^a^)	86.29 (9.80)	84.47 (11.13)	81.33 (12.60)	<0.001^*^	84.20 (10.84)	83.44 (12.31)	83.79 (13.14)	0.574
Influence at work (Mean, SD^a^)	69.07 (20.45)	64.35 (19.87)	58.98 (21.60)	<0.001^*^	64.97 (20.34)	61.54 (22.51)	64.21 (22.28)	0.071
Current smokers %	34.4%	32.5%	37.1%	0.237	38.8%	28.7%	32.6%	0.029^*^
Workplaces %								
Elder care Center	42.5%	41.8%	39.2%	0.411	37.0%	42.1%	37.9%	0.247
Home care	29.7%	33.8%	30.6%		25.7%	28.8%	32.6%	
Hospital	6.8%	3.4%	7.1%		6.3%	6.5%	1.5%	
Other	21.0%	21.1%	23.1%		31.0%	22.6%	28.0%	

^*^ p < 0.05

^a^ Standard Deviation.

Table 2 shows the results of the prospective analyses of the association between physical work load, body weight and the risk of developing LBP. In the unadjusted analyses (Model I), compared with health care workers with low physical work load, those with high physical work load had a significantly higher risk for developing LBP 1–7 days (OR 2.02), as well as LBP over 7 days (OR 2.16) than health care workers with low physical work load. After adjustment for possible confounders (Model II), the health care workers with high physical work load had a 78% increased risk for LBP 1–7 days (OR 1.78), as well as LBP over 7 days (OR 1.78) compared to their colleagues with low physical workload. In both the unadjusted and adjusted analyses, overweight and obese health care workers did not have a significant increased risk for developing LBP compared to their normal weighted colleagues.

**Table 2:** Multinomial logistic regression model estimating the predictive effect (OR and 95% CI) of levels of BMI and levels of physical work load on the development of low back pain

Low back pain 1–7 days						
	BMI			Physical work load		
	Normal	Overweight	Obese	Low	Moderate	High
Model I ^a^	Ref.	0.95/|0.59-1.51|	0.91/|0.50-1.68|	Ref.	1.43/|0.85-2.39|	2.02*/|1.23-3.33|
Model II ^b^	Ref.	1.05/|0.65-1.70|	0.92/|0.49-1.73|	Ref.	1.25/|0.74-2.12|	1.78*/|1.06-2.98|
Low back pain over 7 days						
	BMI			Physical work load		
	Normal	Overweight	Obese	Low	Moderate	High
Model I ^a^	Ref.	1.07/|0.72-1.60|	0.81/|0.46-1.42|	Ref.	1.35/|0.85-2.13|	2.16*/|1.39-3.34|
Model II ^b^	Ref.	1.09/|0.72-1.65|	0.79/|0.45-1.41|	Ref.	1.18/|0.74-1.89|	1.78*/|1.13-2.80|

## Discussion

The main findings of this study among newly educated health care workers without prior history of LBP were that overweight and obese female health care workers were not at increased risk for LBP two years after graduation. On the other hand, female health care workers with high physical work demands were at increased risk for LBP. As our study was conducted on respondents without prior history of LBP, the results support the notion that BMI and LBP are not causally related, thereby lending credence to previous studies not finding an association between BMI and LBP [21-23] .

Our study is the first to investigate the effect of overweight and obesity on the development on LBP among newly educated female health care workers without prior history of LBP. However, another study by Van Nieuwenhuyse et al. look into the effects of physical characteristics measured in physical examinations on the development of LBP among health care workers and distribution companies with no or little LBP history [24]. That study finds a significant effect of obesity on later development of LBP among asymptomatic health care workers. However, their results are not directly comparable to ours because their analysis did not control for work environment factors. Although overweight and obesity do not seem to be risk factors for developing LBP among health care workers, they were shown to be related with the development of hypertension, coronary heart disease, type 2 diabetes and stroke [25]. Notably, 41% of female health care workers were overweight in our study population. Thus, from a public health perspective, initiatives for reducing overweight and obesity among health care workers are recommended.

Female health care workers with high physical work load had 78% increased risk for developing LBP compared to their colleagues with low physical work load. This finding validates previous observations among health care workers [11-13,26], although neither of these included newly educated female workers without prior history of pain. Our unique design provides strong evidence that high physical work load increases the risk for the development of LBP among health care workers. The strength of our study is the baseline population without prior exposure to health care work and without a life-time history of LBP, followed prospectively for two years into their working life after graduation. This is particularly important given that a previous study has indicated that LBP and disability due to LBP during baseline is a significant indicator for dropout in the following two years [9]. As shown in Table 1, there was no difference among respondents with normal weight, overweight and obese concerning their estimated physical workload. As many respondents may have been overweight or obese for several years, a concern is the selection of overweight and obese individuals without LBP in the analysis. An additional analysis showed no differences in prevalence of LBP between normal weight (60%) and overweight (58%) respondents at baseline, whereas obese showed a significantly increased prevalence of LBP (65%, P < 0.001). However, the cross-sectional nature of this baseline correlation does not reveal anything about causality. Because previous episodes of LBP have been shown to be a risk factor for new episodes of LBP [14,27], the exclusion of respondents with prior history of LBP strengthens the validity of our findings. Self-administered questionnaires have been criticized for not providing accurate information on exposure to physical work load. However, persons with LBP may tend to perceive their physical workload as higher than those without LBP [28,29], bringing the results that include those with previous LBP to question [11-13,26,30]. As the respondents included in our study had no prior history of LBP, a systematic bias on the reported physical work load is not very likely.

Recall bias is a limitation of questionnaire research. Thus, some people may have forgotten a previous experience of LBP. Because the mean age of the newly educated health care workers was 35 years, many may have had experience working in other jobs and have forgotten periods of LBP. Thus, we controlled for age in the analyses. Finally, other factors like job satisfaction and education that have not been included in our analyses could be confounders in the relationship between physical work load and BMI and LBP.

## Conclusion

This study showed that overweight and obesity do not increase the risk for developing LBP among newly educated female health care workers. By contrast, new episodes of LBP were related to the level of physical work load among newly educated female health care workers. Thus, prevention of LBP among health care workers ought to focus on alleviating the high physical work loads related to those jobs.

## Competing interests

The authors declare that they have no competing interests

## Authors’ contributions

All authors have made substantial contributions to conception and design of this study. Author JNJ and TCL contributed to collection of data. Author JNJ performed the statistical analyses and wrote the draft of the paper with significant feedback and revision of intellectual content from all co-authors. All authors have read and approved the final version.

## Pre-publication history

The pre-publication history for this paper can be accessed here:

http://www.biomedcentral.com/1471-2474/13/87/prepub
